# The immunosuppressive effect of the tick protein, Salp15, is long-lasting and persists in a murine model of hematopoietic transplant

**DOI:** 10.1038/s41598-017-11354-2

**Published:** 2017-09-06

**Authors:** Julen Tomás-Cortázar, Itziar Martín-Ruiz, Diego Barriales, Miguel Ángel Pascual-Itoiz, Virginia Gutiérrez de Juan, Alfredo Caro-Maldonado, Nekane Merino, Alberto Marina, Francisco J. Blanco, Juana María Flores, James D. Sutherland, Rosa Barrio, Adriana Rojas, María Luz Martínez-Chantar, Arkaitz Carracedo, Carolina Simó, Virginia García-Cañas, Leticia Abecia, José Luis Lavín, Ana M. Aransay, Héctor Rodríguez, Juan Anguita

**Affiliations:** 1 CIC bioGUNE, 48160 Derio, Bizkaia Spain; 2 Ikerbasque, Basque Foundation for Science, 48013 Bilbao, Bizkaia Spain; 3 Department of Animal Medicine and Surgery, School of Veterinary Medicine, Complutense University of Madrid, 28040 Madrid, Spain; 4 Centro de Investigación Biomédica en Red de enfermedades hepáticas y digestivas (CIBERehd), Instituto de Salud Carlos III, 28029 Madrid, Spain; 5 Centro de Investigación Biomédica en Red en cáncer (CIBERonc), Instituto de Salud Carlos III, 28029 Madrid, Spain; 6 Department of Biochemistry and Molecular Biology, University of the Basque Country, 48940 Leioa, Bizkaia Spain; 7 Molecular Nutrition and Metabolism, Institute of Food Science Research (CIAL, CSIC), 28049 Madrid, Spain

## Abstract

Salp15, a salivary protein of *Ixodes* ticks, inhibits the activation of naïve CD4 T cells. Treatment with Salp15 results in the inhibition of early signaling events and the production of the autocrine growth factor, interleukin-2. The fate of the CD4 T cells activated in the presence of Salp15 or its long-term effects are, however, unknown. We now show that Salp15 binding to CD4 is persistent and induces a long-lasting immunomodulatory effect. The activity of Salp15 results in sustained diminished cross-antigenic antibody production even after interruption of the treatment with the protein. Transcriptionally, the salivary protein provokes an acute effect that includes known activation markers, such as *Il2* or *Cd44*, and that fades over time. The long-term effects exerted by Salp15 do not involve the induction of either anergy traits nor increased populations of regulatory T cells. Similarly, the treatment with Salp15 does not result in B cell anergy or the generation of myeloid suppressor cells. However, Salp15 induces the increased expression of the ectoenzyme, CD73, in regulatory T cells and increased production of adenosine. Our study provides a profound characterization of the immunomodulatory activity of Salp15 and suggests that its long-term effects are due to the specific regulation of CD73.

## Introduction

Tick saliva is composed of a cocktail of pharmacologically active biomolecules that modulate among other host responses, the activation of CD4 T cells^1–5^. One of the best-studied components of tick saliva is the protein Salp15 from *Ixodes scapularis*. This protein inhibits the activation of naïve CD4 T cells, blocking early T cell signaling pathways, and thereby, the production of the autocrine growth factor IL-2^6–10^. Salp15 specifically binds to the first domain (D1) of the CD4 co-receptor causing a conformational change that impedes its interaction with the Src kinase, Lck and its activation^11^. Due to the specificity of Salp15 for CD4 and its capacity to inhibit the activation of CD4 T cells, the use of this tick saliva protein has been suggested for the treatment of immune diseases. In addition, our group has demonstrated the ability of Salp15 to inhibit the development of CD4 T cell-mediated immune responses *in vivo* upon challenge with different antigens^7, 10, 12^. Moreover, Salp15 prevents the development of experimental asthma in a mouse model^7^. However, whether the inhibition of CD4 T cells by Salp15 has long-term effects on future encounters with specific or unrelated antigens is currently unknown.

A common complication associated with allogeneic hematopoietic stem cell transplantation (HSCT) is the appearance of Graft-versus-host disease (GvHD). GvHD appears when donor T cells recognize as foreign the recipient antigens, including both human leukocyte (HLA) and minor histocompatibility antigens. Allogeneic HSCTs are used both in treatments of malignant disease and in ordinary transplants. GvHD appears in 50% of the transplants and causes death in 15% of the cases^13^. Despite its effectiveness, the induction of immunosuppression after HSCT can produce undesirable effects. These include the inhibition of graft-versus-tumor effector cells (GvT) and the appearance of infections and neoplasms^13–17^. Other treatments used for the mitigation or elimination of this disease are ineffective and unspecific. In fact, pre-transplantation chemotherapy and radiotherapy treatments (conditioning) applied in these cases for the elimination of the cancer cells and the establishment of the transplanted cells can result in nonspecific inflammatory events, helping create the necessary conditions for the activation of donor T cells^18^. Although several murine models of transplantation exist^19, 20^, none recapitulates in full the pathology observed in human transplantation. The transplant model of pure strains into F1 offspring does not require previous conditioning and results in mild episodes of acute GvHD followed by a period of chronic disease characterized by the production of autoantibodies^21^.

Because Salp15 is able to inhibit early T cell signaling events, we hypothesized that the protein could preclude the activation of CD4 T cells and induce a long-term unresponsive or anergic after the exposure to the salivary protein. Our results show that Salp15 is able to change the transcriptional program of CD4 T cells during activation that nevertheless fades over time and does not result in increased populations of anergic or regulatory T cells. However, the protein induces the upregulation of the ectoenzyme, CD73 on the surface of Tregs, inducing increased production of the immunosuppressive molecule adenosine. Overall, the activity of Salp15 is evident in a long-term transplantation murine model and prevents the deposition of immune complexes in the kidney, a hallmark of murine chronic GvHD^21^.

## Results

### The effect of Salp15 on activating CD4 T cells is long-lasting

**Figure 1:**
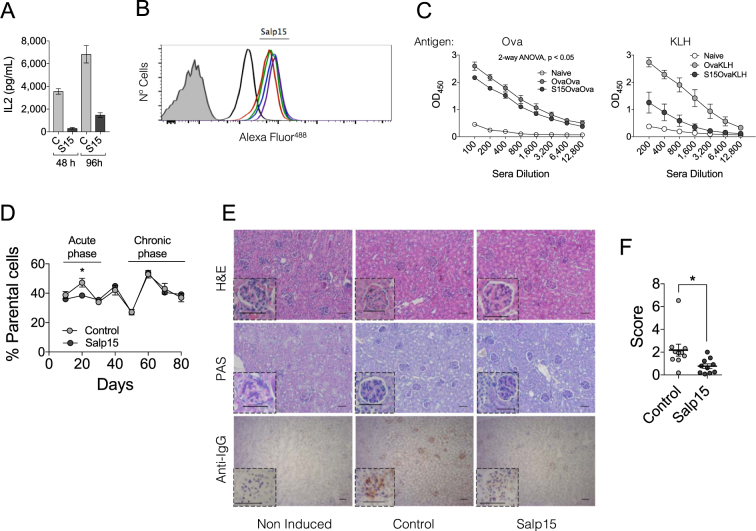
The effect of Salp15 on CD4 T cells is long-lasting. (**A**) IL-2 production by purified splenic CD4 T cells activated with anti-CD3/CD28 for 2 days in the presence of 50 μg/ml of Salp15, washed and re-stimulated under the same conditions for another 48 h in the absence of the immunosuppressive protein. The results represent the average ± SE of one experiment in triplicate and are representative of 3 performed. (**B**) Binding of Salp15 or the C-terminal deletion mutant, Salp15ΔP11 to purified CD4 T cells. The proteins were labeled with Alexa Fluor^488^ and tested for their binding for different time periods. The binding was assessed by flow cytometry. The shaded histogram represents unlabeled cells. The black histogram represents binding of Salp15ΔP11. Color histograms represent binding of Salp15 at different time points (12, 24, 48 and 72 h). (**C**) Antibody titers specific for ovalbumin (left panel) and KLH (right panel) in mice immunized with the antigens as described in Methods and treated with 50 μg of Salp15 (S15OvaOva or S15OvaKLH) or left untreated (OvaOva or OvaKLH). Non-immunized mice served as controls (Naive) (**D**) Percentage of parental (H2^b^) cells in the blood of transplanted CB6F1 mice (H2^b,d^) over a period of 80 days. The mice were either treated with 50 μg of Salp15 or Salp15ΔP11 (Control) (**E**) Histological features of the kidneys of CB6F1 mice transplanted with B6 splenocytes after 80 days. Kidney sections were stained with H&E (top panels), periodic acid schiff (PAS) staining (middle panels) and anti-mouse IgG immune complex deposition (lower panels). The scale bars represent 50 μm. (**F**) Assessment of IgG immune complex deposition scores in the kidneys of the transplanted mice by analysis of 5 different micrographs with the FriDA software package and averaged per section. The experiments *in vivo* were performed with groups of 5 mice and performed at least twice.

In order to determine whether the effect of Salp15 on the activation of CD4 T cells is sustained, we activated purified splenic CD4 T cells in the presence of the salivary protein for 2 days, followed by their extensive washing and re-stimulation for 2 more days. The production of IL-2 was significantly reduced at both time points, including after 4 days of activation when Salp15 was no longer present (Fig. 1A). The longer-term effect of Salp15 could be due to its persistent binding to the surface of CD4 T cells. Thus, we determined the binding of Alexa Fluor^488^-labeled Salp15 as well as the inactive control (Salp15ΔP11) by flow cytometry. Although both Salp15 and Salp15ΔP11 bound to purified CD4 T cells, the deletion of the C-terminal peptide, P11, resulted in decreased binding (Fig. 1B) in agreement with its reported lack of activity^22^. Importantly, binding of Salp15 to CD4 T cells was detectable for up to 72 h (Fig. 1B), indicating a persistent ability of this protein to remain attached to CD4.

In order to assess whether Salp15 could also exert long-term effects *in vivo*, we performed an immunization regime against ovalbumin and the unrelated protein, keyhole limpet hemocyanin (KLH). Groups of mice were immunized with ovalbumin in aluminum hydroxide in the absence or presence of Salp15 (day 0). Seven days later, the mice were boosted with ovalbumin under the same conditions. At day 14, the mice in each group were subdivided and immunized with ovalbumin or KLH in the absence of Salp15. All the mice were sacrificed at day 21. Sera were then analyzed for the presence of ovalbumin- and KLH-specific IgG levels. As expected, ovalbumin-specific IgG levels were significantly lower in mice that had received Salp15 in the first 2 immunizations (Fig. 1C). In order to establish whether the effect of Salp15 was circumscribed to ovalbumin, we also determined sera IgG levels against KLH. Mice that had not been immunized with this antigen did not show KLH-specific IgG in the sera. Notably, the levels of KLH-specific IgG were high in those mice receiving the antigen, and significantly lower in those that had received two previous doses of Salp15, but not at the time of immunization with KLH (Fig. 1C). These data show that the effect of Salp15 lasts beyond the treatment and can affect the response to unrelated antigens, such as KLH.

To further address the long-term effect associated with the treatment with Salp15, we utilized a murine model of GvHD resulting from the adoptive transfer of spleen cells from B6 into unconditioned CB6 F1 mice^21^. In this model of transplantation, the proliferation of drafted cells can be monitored in the blood and results in two different phases of the disease: an acute phase in which the transplanted cells proliferate, followed by a chronic phase predominantly characterized by symptomatology similar to autoimmune disease, including the deposition of IgG immune complex in the kidney^21^. CB6 F1 mice were transplanted 60 × 10^6^ spleen cells and divided into two groups. One of the groups received intraperitoneal injections of Salp15 every other day for 10 days, while the control group received the same dose of Salp15ΔP11. Control animals showed a peak of parental cells at day 20 relative to the transplant, followed by a decline and another increase around 60 days post-transplant (Fig. 1D). In contrast, the treatment with Salp15 during the first 10 days post transplantation resulted in a significantly reduced level of parental cells in the blood (Fig. 1D). However, no effect was detected during the chronic phase of the disease. We also analyzed the potential effect of the treatment with Salp15 on the pathology associated with this model at day 80 after transplantation of the parental splenocytes. CB6 F1 mice transplanted with B6 splenocytes showed some disorganization in the glomerular basement membrane and tubular brush border of the kidney upon transplantation that was not affected by the treatment (Fig. 1E). However, immune complex deposition that was readily detected in the control-treated mice was significantly reduced in the animals that had been treated with Salp15 (Fig. 1E,F). Overall, our results show that Salp15 has short-term and long-term effects during the development of immune responses that specifically affect the expansion of CD4 T cells and the production of antibodies.

### Identification of transcriptional traits in activating CD4 T cells treated with Salp15

**Figure 2:**
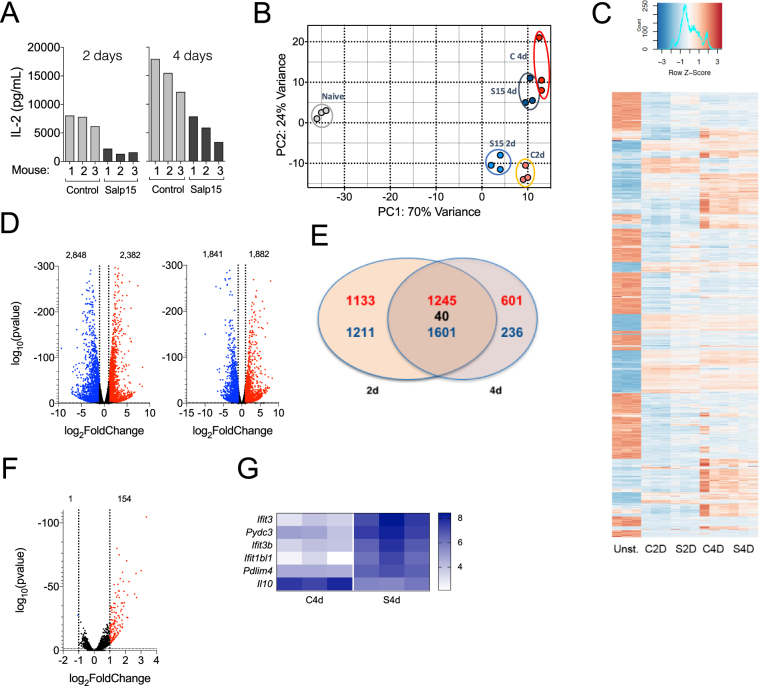
Transcriptional traits of CD4 T cells activated in the presence of Salp15. (**A**) IL-2 production by CD4 T cells used for the transcriptomic analysis. Each bar represents one of the 3 mice used. The amount of Salp15 used was 25 μg/ml. Control cells were treated with Salp15ΔP11 (Control). (**B**) Principal component analysis showing the grouping of the different assay conditions according to their transcriptome. Non-activated cells (Naive, grey); Salp15ΔP11-treated at 2 days (C 2d, orange) or 4 days of activation (C 4d, red); Salp15-treated at 2 days (S15 2d, light blue) or 4 days (S15 4d, dark blue). (**C**) Heatmap corresponding to the 1000 most regulated genes over the conditions analyzed by RNAseq. (**D**) Volcano plots showing the genes upregulated (red) or downregulated (blue) by activation with anti-CD3 and CD-28 at 2 (left) and 4 days (right) of stimulation. (**E**) Venn diagram showing the number of genes regulated at both 2 and 4 days of activation in the absence of Salp15. (**F**) Volcano plot showing the number of genes differentially regulated during the activation of CD4 T cells in the presence of Salp15 or Salp15ΔP11 (control) after 2 days of stimulation. (**G**) Heatmap of the genes regulated in the presence of Salp15 at 4 days of activation.

We then sought to determine the transcriptional signature of activated CD4 T cells for 48 and 96 hours with plate-bound anti-CD3 and soluble anti-CD28 in the presence of Salp15 or its control, Salp15ΔP11 (Fig. 2A). To allow the survival of the CD4 T cells throughout the activation process, a low dose of Salp15 (25 μg/ml) was used. This dose reduced the activation of CD4 T cells (Fig. 2A) without significant cell death at 4 days post-activation (data not shown). Principal component analysis (PCA) showed a distinct pattern of gene expression in control activated cells over the analyzed period of time (Fig. 2B). The presence of Salp15 resulted in variations in the PCA at 2 days of activation, while the differences faded when the transcriptome was analyzed after 4 days of activation (Fig. 2B). These differences were also noticeable when the 1000 most regulated genes were analyzed, with maximal differences between Salp15-treated and control activated CD4 T cells at 2 days and more discrete differences when analyzed at 4 days of activation (Fig. 2C). The activation of CD4 T cells under control conditions revealed 2382 genes upregulated and 2848 genes downregulated at 2 days of activation (Fig. 2D), while 1882 genes were upregulated and 1841 genes downregulated after 4 days of activation, using a cut-off value of 1 log_2_fold change and an adjusted p-value < 0.05 (Fig. 2D). Of these genes, 1245 were upregulated at both 2d and 4d of activation, 1601 were downregulated at both time points and 40 were regulated in opposite directions (Fig. 2E). Using the same cut-off values, we found 154 genes upregulated as a consequence of the presence of Salp15 during activation at 2d while only 1 gene was downregulated (Fig. 2F). Notably, the number of genes regulated at 4d of activation in the presence of Salp15 was dramatically reduced to 5 genes upregulated and just 1 (*Il10)* downregulated (Fig. 2G). Selected transcriptional changes were validated as shown in Supplementary Fig. 1.

### Salp15 affects CD4 T cell genes early during the activation process

**Figure 3:**
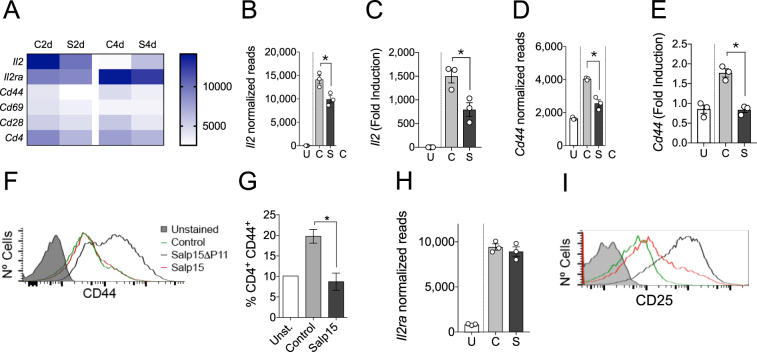
Salp15 affects the activation of CD4 T cells. (**A**) Heatmap of genes involved in the activation of CD4 T cells at 2 and 4 days of stimulation with anti-CD3/CD28 in the absence or presence of Salp15. (**B**) Normalized reads corresponding to the *Il2* gene at 2 days of activation in the presence of Salp15 (S) or Salp15ΔP11 (**C**). U- Unstimulated cells. (**C**) Transcriptional levels of *Il2* at 2 days of activation in the presence of Salp15 (S) or Salp15ΔP11 (**C**) of Salp15 determined by qRT-PCR. Normalized reads of *Cd44* (**D**) and transcriptional levels by qRT-PCR (**E**) in CD4 T cells at 2 days of activation, as before. (**F**) Surface CD44 levels on CD4 T cells activated for 2 days in the presence of Salp15 or Salp15ΔP11. Control: Unstimulated cells. The grey histogram represents an unstained control. (**G**) Percentage of CD4 T cells expressing CD44 in the spleen of mice immunized with ovalbumin and treated with Salp15 compared to control treated, immunized mice (Control) and non immunized mice (Unst.). (**H**) Normalized reads of *Il2ra* in CD4 T cells at 2 days of activation, as before. (**F**) Surface CD25 levels on CD4 T cells activated for 2 days in the presence of Salp15 or Salp15ΔP11. The color legend is as indicated in (**F**). All the experiments were performed with 25 μg/ml of Salp15 or Salp15ΔP11 (control).

The activation of CD4 T cells produced the expected profile, involving genes such as *Il2*, *Cd44* or *IL2ra* (Fig. 3A). Gene ontology analysis of Biological Processes (GOBP) revealed that the most over-represented groups included genes related to leukocyte cell-cell adhesion and aggregation or T cell activation, among other immune-related processes (Supplementary Fig. 2). As expected, the presence of Salp15 induced a reduction of *Il2* gene expression and the production of IL-2 at 2 days of activation (Fig. 3A,B,C; see also Fig. 2A). Furthermore, the activation marker CD44 was significantly reduced both at the gene expression level (Fig. 3A,D,E), by surface analysis of the protein by flow cytometry in *in vitro* activated CD4 T cells (Fig. 3F), and *in vivo* in the spleens of ovalbumin-immunized mice at day 7 post-immunization (Fig. 3G). Although, the effect of Salp15 on *Il2ra* expression was not evident at this concentration (25 μg/ml, Fig. 3H), the analysis of CD25 on the surface of 2-day activated CD4 T cells revealed decreased levels of the protein in the presence of this dose of Salp15 (Fig. 3I).

### Salp15 induces the expression of 5′-ectonucleotidase (CD73) in regulatory T cells

**Figure 4:**
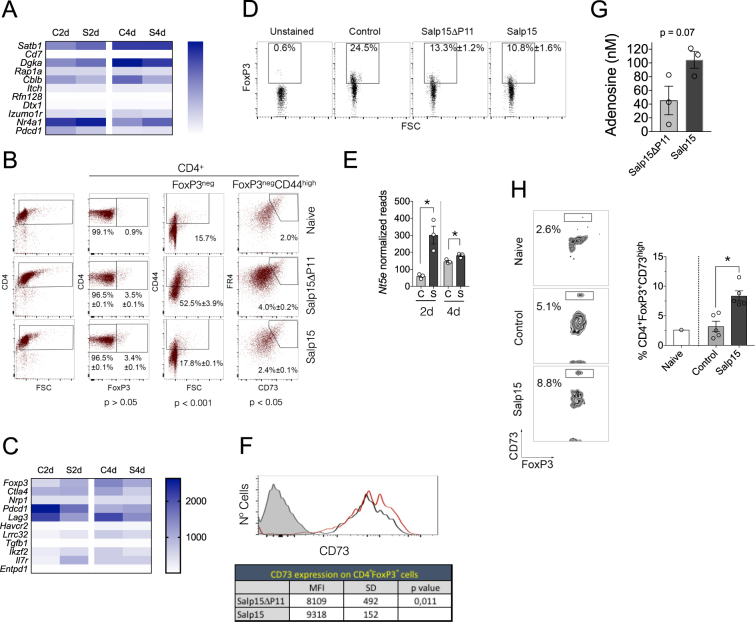
Salp15 does not induce increased anergic or Treg numbers but augments the levels of CD73 in FoxP3^+^ cells. (**A**) Heatmap representing genes associated with anergy in CD4 T cells according to the RNAseq analysis in the presence of Salp15 (S) or Salp15ΔP11 (**C**) at 2 and 4 days of activation. (**B**) Percentage of FoxP3^+^ and anergic CD4 T cells upon their exposure to 25 μg/ml of Salp15 or Salp15ΔP11 for 2 days. The average ± SE of triplicates is indicated. The p values (Student´s t test) correspond to the comparison between the Salp15ΔP11 and Salp15 groups. The data presented is representative of 2 independent experiments with similar results. (**C**) Heatmap corresponding to genes associated with regulatory T cells according to the RNAseq analysis in the presence of Salp15 (S) or Salp15ΔP11 (**C**) at 2 and 4 days of activation. (**D**) Percentage of FoxP3-positive CD4 T cells upon exposure to 25 μg/ml of Salp15 or Salp15ΔP11 *in vitro* for 2 days during activation. The values correspond to the average ± SE of 5 mice per group. No significant differences were detected between the Salp15ΔP11 and Salp15 groups. The data is representative of 2 independent experiments. (**E**) Normalized reads corresponding to the expression levels of *Nt5e* at 2 and 4 days of activation *in vitro* in the presence of Salp15 (S) or Salp15ΔP11 (**C**). (**F**) Surface expression levels of CD73 on CD4^+^FoxP3^+^ T cells (top) activated for 2 days in the presence of 25 μg/ml of Salp15 (red histogram) or Salp15ΔP11 (black histogram). The shaded histogram represents an unstained control. The table shows the average mean fluorescence intensity (MFI) ± standard deviation (SD) of 5 mice per group. Differences of the means were analyzed by the Student´s T-test. (**G**) Average adenosine levels in the supernatants of CD4 T cells activated in the presence of 25 μg/ml of Salp15 or Salp15ΔP11 for 2 days. The results represent 3 independent mice. (**H**) Increased percentage of CD4^+^FoxP3^+^CD73^high^ cells in the blood of CB6F1 mice transplanted with B6 splenocytes at day 50 post induction. The histogram on the right represents the average ± SE of 5 control (Salp15ΔP11) and 5 Salp15-treated mice. *p < 0.05.

The repressed activation of CD4 T cells in the presence of Salp15 could result in the induction of anergy. We therefore analyzed the expression levels of genes associated with this phenomenon in CD4 T cells, including *Satb1*, *Cd7*, *Rap1a*, *Itch*, *Rnf128*, *Dtx1*, *Izumo1r*, *Cblb, Dgka* ^23^, *Nr4a1* or *Pdcd1* ^24^. Salp15 induced small and inconsistent changes in these genes (Fig. 4A, Table 1), suggesting that this protein does not induce anergy in CD4 T cells. In order to confirm these results, we analyzed by flow cytometry markers of anergy in CD4 T cells that were activated *in vitro* in the presence of Salp15. The percentage of CD4^+^FoxP3^−^CD44^high^CD73^high^FR4^high^ ^[ 24^ cells increased upon CD4 T cell activation but remained as low as in naive cells in the presence of Salp15 (Fig. 4B). Furthermore, the analysis of anergic CD4 T cells in mice either immunized with ovalbumin (Table 2) or induced GvHD (Table 3) showed no effect on this population as a consequence of the treatment with Salp15. Similarly, the treatment with Salp15 did not result in the generation of a significant anergic B cell population or increased the pool of monocytic myeloid suppressor cells (Table 2). These data confirm that Salp15 act as an immunosuppressor on CD4 T cells that depends on its interaction with CD4 and that does not induce a long-term anergic state in T or B cells, nor the generation of myeloid suppressor cells.

**Table 1:** Differential expression of genes associated with CD4 T cell anergy and regulatory T cells.

Gene	S2D vs. C2D		S4D vs. C4D	
	Log_2_FI	Adj. p-value	Log_2_FI	Adj. p-value
Anergy				
*Satb1*	0,304	0,012	0,002	0,997
*Cd7*	0,750	0,001	0,166	NA
*Rap1a*	0,030	0,894	−0,223	0,116
*Itch*	0,087	0,613	−0,065	0,816
*Rnf128*	−0,077	0,858	−0,321	0,186
*Dtx1*	−0,039	0,908	0,047	0,921
*Izumo1r*	−0,090	0,573	−0,251	0,094
*Cblb*	−0,403	8,40E-06	−0,656	3,74E-07
*Dgka*	0,282	0,001	−0,215	0,114
*Nr4a1*	0,148	0,189	0,426	1,1E-04
*Pdcd1*	−0,568	3,9E-11	−0,144	0,612
Regulatory T cells				
*Foxp3*	0,815	7,85E-09	−0,352	0,095
*Ctla4*	0,109	0,535	−0,691	1,19E-07
*Nrp1*	0,048	0,859	0,015	0,975
*Pdcd1*	−0,568	3,90E-11	0,144	0,612
*Lag3*	−0,721	1,81E-11	−0,540	5,44E-07
*Havcr2*	−0,166	0,668	−0,177	0,620
*Lrrc32*	0,527	0,044	−0,268	0,345
*Tgfb1*	−0,148	0,197	−0,164	0,473
*Ikzf2*	0,438	0,117	−0,558	0,00027
*Il7r*	***1,504***	***6,21E-15***	−0,040	0,914
*Entpd1*	0,402	0,163	−0,004	0,995
*Type 1 Regulatory T cells*				
***Il10***	−0,236	0,492	***−1,069***	***4,01E-10***
*Eomes*	0,136	0,477	−0,193	0,351
*Il2rb*	0,171	0,367	−0,069	0,808
*Itga4*	0,536	0,027	−0,260	0,178
*Itgb7*	−0,578	1,78E-06	0,047	0,878
*Ly6c1*	0,200	0,233	−0,190	0,511
*Tigit*	−0,387	0,001	−0,500	0,001

**Table 2:** Cell populations in blood and spleen of mice immunized with ovalbumin (O) and treated with Salp15 (S) or the control protein (Δ).

Blood B cell populations*	Control	ΔO		SO	
		*Average*	*SD*	*Average*	*SD*
B cells (B220^+^)	45,10	43,50	1,87	40,07	4,43
B220^+^CD69^+^	28,50	12,41	4,15	11,50	4,76
Anergic B cells (B220^+^CD93^+^IgM^+^CD23^+^)	3,85	2,28	0,40	1,80	0,76
Mature B cells (B220^+^CD93^−^CD23^+^)	27,87	28,77	0,56	27,80	1,50
Spleen populations*	**Control**	**ΔOO**		**SOO**	
		*Average*	*SD*	*Average*	*SD*
CD4^+^	11,60	11,02	2,31	10,77	2,08
CD8^+^	7,84	6,10	0,98	6,38	0,87
CD4^+^CD25^+^	12,90	8,47	1,33	5,60	2,34
CD4^+^CD44^+^	28,10	7,48	2,43	7,55	4,21
Anergic T cells (CD4^+^CD44^high^CD73^high^FR4^high^)	3,60	0,92	0,35	0,86	0,39
Tregs (CD4^+^NRP1^+^)	2,02	1,54	0,20	1,46	0,16
Macrophages (F4/80^+^GR-1^−^)	2,15	2,08	0,09	2,44	1,30
Neutrophils (F4/80^−^GR-1^+^)	1,30	1,52	0,21	2,73	0,71
Monocytic myeloid suppressor cells (CD11b^high^GR-1^high^Ly6C^+^)	1,01	0,56	0,24	0,82	0,36
Inflammatory macrophages (F4/80^+^GR-1^int^Ly6C^high^)	1,97	2,42	0,38	2,52	0,64
		n = 5		n = 5	

All comparisons between ΔO vs. SO, p > 0.05. *The gating strategies are shown in Supplementary Fig. 3.

**Table 3:** Cell populations (average ± SD) in blood upon B6 splenocyte transplant into CB6 F1 mice.

Blood cell populations	Day 20		Day 30		Day 40		Day 60		Day 80	
	*Control*	*Salp15*	*Control*	*Salp15*	*Control*	*Salp15*	*Control*	*Salp15*	*Control*	*Salp15*
CD4^+^ T cells	26.1 ± 0.7	26.5 ± 0.5	33.5 ± 1.6	30.0 ± 1.2	34.9 ± 2.2	38.4 ± 2.7	30.9 ± 1.6	28.5 ± 1.8	21.0 ± 1.8	25.2 ± 2.7
CD8^+^ T cells	12.8 ± 1.2	13.8 ± 0.4	17.6 ± 1.0	18.0 ± 0.9	18.5 ± 1.1	17.4 ± 0.8	15.6 ± 0.8	16.2 ± 0.4	15.9 ± 1.4	18.4 ± 1.6
Tregs (CD4^+^FoxP3^+^)	7.1 ± 0.4	6.2 ± 0.3	7.3 ± 0.3	7.1 ± 0.2	4.7 ± 0.4	3.9 ± 0.7	6.8 ± 0.4	7.2 ± 0.5	7.7 ± 0.6	7.7 ± 1.2
Anergic T cells (CD4^+^CD44^high^CD73^high^FR4^high^)	6.2 ± 1.8	3.9 ± 0.9	20.0 ± 2.7	16.5 ± 1.9	13.4 ± 1.1	12.3 ± 1.1	4.4 ± 0.9	5.4 ± 0.5	7.0 ± 1.4	5.9 ± 1.0

All comparisons between Control vs. Salp15, p > 0.05. n = 5 mice per group.

The transcriptomic data also helped us elucidate whether activated CD4 T cells in the presence of Salp15 acquired specific markers of regulatory T cells, such as *Foxp3, Ctla4, Nrp1, Pdcd1, Lag3, Havcr2, Lrrc32, Tgfb1, Ikzf2, Il7r* or *Entpd1* ^25, 26^. No major differences were found between controls and CD4 T cells activated in the presence of Salp15 (Fig. 4C, Table 1). Moreover, the analysis of FoxP3-positive cells after 4 days of activation *in vitro* did not show changes in the percentage of Tregs associated with the presence of Salp15 (Fig. 4B). The effect of Salp15 on the pool of Tregs *in vivo* was also negligible in mice that had been immunized with ovalbumin (Fig. 4D). Furthermore, the analysis of FoxP3-positive CD4 T cells in the peripheral blood of CB6 F1 mice transplanted with B6 splenocytes did not show differences associated with the treatment with Salp15 throughout the life of the experiment (Table 3). Similar results were found when we analyzed the expression of Nrp1 on CD4 T cells, another marker of regulatory T cells^27^ (Table 2). The analysis of type 1 regulatory T (Tr1) cells markers, including *Il10*, *Eomes*, *Il2rb*, *Itga4*, *Itgb7*, *Ly6c1* or *Tigit* ^28^, showed that Salp15 does not induce the generation of these cells, although *Il10* expression levels were significantly reduced in the presence of the salivary protein at 4 days of activation (Table 1). These results demonstrate that the treatment with Salp15 does not result in the generation of a population of regulatory T cells that could account for long-term immunomodulatory effects.

We then addressed whether the treatment with Salp15 would impact the activity rather than the size of the Treg population. The ectoenzyme CD73 is expressed by Tregs and mediates the production of adenosine, an immunosuppressive molecule on T cells^29–31^. We observed that expression levels of *Nt5e* (which encodes CD73) were increased upon the treatment of CD4 T cells with Salp15 (Fig. 4E). Therefore, we determined the expression levels of CD73 on the surface of FoxP3-positive cells. We found that the activation of CD4 T cells *in vitro* in the presence of Salp15 resulted in a significant increase in the surface expression of CD73 (Fig. 4F). Furthermore, the levels of adenosine increased upon the treatment of activating CD4 T cells with Salp15 (Fig. 4G). Importantly, we observed an increase in a population of CD4^+^FoxP3^+^CD73^high^ in the blood of mice that have been induced GvHD at day 50 post transplant (Fig. 4H). Overall, our results show that Salp15 is able to induce long-term effects on activating CD4 T cells that involve, at least in part, the increased expression and activity of CD73 on regulatory T cells.

## Discussion

The tick salivary protein Salp15 inhibits early CD4 T cell signaling events and, in consequence, their activation. The activity of Salp15 on CD4 T cells is well-characterized^9, 10, 24^. Both *in vitro* and *in vivo*, this protein is able to prevent a full activation program on these immune cells^6^. The fate of CD4 T cells affected by the activity of Salp15 is not known. They could become permanently unable to respond to antigen or, alternatively, revert to a state in which they are amenable to become activated when antigen is present again. Here, we show that the presence of Salp15 during the activation of CD4 T cells results in long-term effects that affect their encounter with new antigens. Using transcriptomics, two models of CD4 T cell activation *in vivo* and the polyclonal activation of these cells *in vitro*, we show that whereas Salp15 does not affect the generation of anergic CD4 T cells or Tregs, it induces the increased expression of the ectoenzyme, CD73, in Foxp3^+^ regulatory T cells. This effect results in an elevated production of adenosine, a known immunomosuppressive molecule produced by Tregs^32, 33^.

Treatment with Tregs has been proposed as an appropriate therapy for GvHD and other immune disorders^34, 35^. In fact, several biological agents, including CTLA4 fusion proteins or anti-TNF antibodies^34^, have the ability to either induce an increase in number or the activity of Tregs. Treg function involves CD73 activity and other mechanisms of action^36, 37^. Indeed, the importance of CD73 activity has been studied in a murine model of GvDH, demonstrating that the ectonuclease helps control the disease^30^. The conversion of 5´-AMP to adenosine mediated by CD73 in Tregs has been broadly described^38^. The anti-inflammatory effect of adenosine present in the pericellular microenvironment results in the suppression of proliferation of effector CD4 T cells and the reduction of cytokine production^39^. Indeed, we found that the levels of this molecule were increased in *in vitro* assays of CD4 T cell activation in the presence of Salp15. The capacity of Salp15 to increase *Nt5e* transcription and the upregulation of CD73 on the surface of FoxP3^+^ T cells can therefore help explain the long-lasting effects elicited by Salp15. Future studies will determine whether the absence of CD73 on Tregs can indeed suppress the long-term immunomodulatory effect exerted by the salivary protein.

Our results show that the binding of Salp15 to CD4 persists along the activation period *in vitro*. However, the changes induced by Salp15 at the transcriptional level fade over time. Furthermore, Salp15 does not affect CD4 T cell differentiation in the absence of polarizing cytokines, including the induction of Th1, Th2, Th17 or Treg gene markers (Suppl. Fig. 4A). We cannot exclude that under polarizing conditions, Salp15 may affect CD4 T cell differentiation. In fact, in the presence of IL-6, the inhibition of IL-2 production during CD4 T cell activation can lead to their differentiation towards a Th17 phenotype^22^. Nevertheless, the restimulation of splenocytes of ovalbumin and KLH immunized mice resulted in similar levels of IL-4, while IFNγ was not detected in the restimulation supernatants (Suppl. Fig. 4B), arguing against an effect of Salp15 on Th1 or Th2 differentiation under these conditions. Our analysis also show that Salp15 does not seem to exert a direct effect on other cellular types, including the generation of anergic B cells or myeloid-derived suppressor cells (MDSCs). Nevertheless, in both *in vivo* models, the treatment with Salp15 results in an indirect effect on the ability of B cells to produce antigen-specific antibodies, as we have previously demonstrated^10^.

In summary, we show that the tick salivary immunosuppressor Salp15 is able to induce the sustained repression of CD4 T cell activation that involves the direct effect on these immune cells during the activation period and the increased expression of CD73 on regulatory T cells. These data support the notion that Salp15 is able to maintain its immunomodulatory action through the induction of increased Treg activity, leading to long-term effects in two *in vivo* models, including a pre-clinically relevant murine model of graft versus host disease.

## Methods

### Protein purification and labeling

Salp15 and an inactive deletion mutant lacking the last 20 aminoacids (Salp15ΔP11) were purified from Drosophila S2 cells, as described^10^. Protein labeling was performed using the Alexa Fluor® 488 Protein Labeling Kit (Thermo Fisher Scientific, Eugene, OR), following the manufacturer’s instructions.

### Cell purification and activation

CD4 T cells were purified from the spleens of C57BL/6 mice by negative selection using a CD4 T cell isolation Kit (Miltenyi Biotec, Bergisch Gladbach, GE) according to the manufacturer’s instructions. Purified CD4 T cells at the indicated concentrations were activated with 5 μg/ml of plate-bound anti-CD3ε and 1 μg/ml of soluble anti-CD28 (BD Biosciences, San Diego, CA) in the presence of the indicated concentration of Salp15 or Salp15ΔP11. Cells were incubated at 37 °C in TexMACS Medium (Miltenyi Biotec).

### Flow cytometry

Blood was extracted from the saphenous vein in the presence of EDTA and depleted of erythrocytes by hypotonic lysis. Whole splenocytes were isolated from immunized or GvHD mice by mechanical disruption followed by lysis of erythrocytes. The cells (10^6^/ml) were incubated with Fc Block (anti-CD16/CD32; BD BioSciences) and labelled with fluorochrome-labeled antibodies against CD4, CD8, B220, CD69, Ly6C, F4/80, GR-1, CD25, CD11b, CD44, CD73, FR4, NRP1, Foxp3, CD93, IgM, CD23 (Miltenyi Biotec). To detect Salp15 binding to CD4 T cells, splenic purified CD4 T cells were labeled with Salp15-Alexa Fluor^488^ or Salp15ΔP11-Alexa Fluor^488^.

### Immunizations

Groups of C57Bl/6 mice were immunized subcutaneously with 50 μg of ovalbumin in aluminum hydroxide (50% Hydrogel, Invivogene. Tolouse, France) containing 50 mg of Salp15 or PBS (conbtrol). The mice were boosted under the same conditions 7 days later. At day 14, each group of mice was subdivided into 2 groups and immunized with either 50 μg of ovalbumin or keyhole lympet hemocyanin (KLH). The mice were sacrificed 7 days later and the spleens and sera were analyzed for cellular composition and antigen-specific immunoglobulin titers, respectively.

### Graft versus host disease murine model

Splenocytes were extracted from 8-week old C57BL/6 (H-2^b^) mice and 60 × 10^6^/mouse were injected intraperitoneally into CB6F1 (H-2^b,d^) mice (Envigo, Gannat, France). The mice were treated with 50 μg of Salp15 by intraperitoneal injection starting the day of cell transfer and every other day until day 10. Blood was extracted from day 10 to day 80 at 10-days intervals. Erythrocytes were removed by hypotonic lysis and the cells were analyzed by flow cytometry. At sacrifice, kidneys were processed for histochemical evaluation.

### Renal deposited IgG detection, PAS and HE

Kidneys were fixed in 10% neutral buffered formalin, dehydrated, embedded in paraffin and cut into 5 μm thick sections. For histopathology, sections were hydrated and stained with hematoxylin – eosin (HE) or periodic acid-Schiff (PAS) according to standard protocols. For immunohistochemical analysis, tissue sections were subjected to antigen retrieval using protease K for 20 min at 37 °C. After blocking, sections were incubated with primary antibody overnight. The slides were then sequentially incubated with DAB chromogen for 5 min, counterstained with Mayer’s hematoxylin and mounted for microscopy. Goat Anti-Mouse IgG-HRP Light chain specific (Jackson ImmunoResearch Laboratories) was used at 1/250 concentration as the primary antibody. Photographs were taken with an Axioimager A1 microscope and analyzed with Frida software^40^.

### RNAseq

Purified CD4 T cells from three mice were activated independently with 5 μg/ml of plate-bound anti-CD3 and 1 μg/ml of soluble anti-CD28 (BD Bioscience) in the presence of 25 μg/ml of Salp15 or Salp15ΔP11 (control). Cells were incubated at 37 °C in TexMACS Medium (Miltenyi Biotec) for 48 and 96 h. RNA extraction was performed using the PureLink RNA Micro Scale Kit (Thermo Fisher Scientific) according to the manufacturerʼs protocol. The quantity and quality of the RNAs were evaluated using the Qubit RNA Assay Kit (Invitrogen, Eugene, OR) and RNA Nano Chips in a 2100 Bioanalyzer (Agilent Technologies, Waldbronn, GE), respectively. Libraries for sequencing were prepared using the TruSeq RNA Sample Preparation Kit v2 (Illumina Inc, San Diego, CA) following the protocol provided by the manufacturer. Single-read, 50 nt sequencing of pooled libraries was carried out in a HiScanSQ platform (Illumina Inc.).

The quality control of the sequenced samples was performed with FASTQC software (www.bioinformatics.babraham.ac.uk/projects/fastq). Reads were mapped against the mouse (*mm10*) reference genome by using the program Tophat^41^ to account for spliced junctions. The resulting BAM alignment files for the samples were the input for the Differential Expression (DE) analysis, carried out by DESeq. 2^42^, to detect differentially expressed genes among the different conditions. GO enrichment was tested using the ClusterProfiler^43^ Bioconductor package and the Panther Database^44^. Transcriptomics data were also analyzed using QIAGENʼs Ingenuity Pathway Analysis (IPA, QIAGEN, Red Wood city, CA).

### Real-time RT-PCR

RNA was reverse transcribed using M-MLV reverse transcriptase (Thermo Fisher Scientific) and random hexamers. Real-time PCR was then performed using SYBR Green PCR Master Mix (Quanta Biosciences, Beverly, MA) on a QuantStudio 6 real-time PCR System (Thermo Fisher Scientific). Fold induction of the genes was calculated using the 2^−ΔΔCt^ method relative to the reference, previously validated genes, *Rpl19* and *Actb*, as indicated. The primers used are listed in Table 4.

**Table 4:** Sequence of the Primers used for RNAseq validation by qRT-PCR.

Gene	Forward	Reverse	Purpose
*IL2*	TGTGCTCCTTGTCAACAGCG	TTTCAATTCTGTGGCCTGCTTG	RNAseq validation
*Zfp750*	CCCACCTTTGCTGCAGGT	GGGGGCCGTGGAATATAGTG	RNAseq validation
*Klf2*	GCCTTCGGTCTTTTCGAGGA	AGGCTTCTCACCTGTGTGTG	RNAseq validation
*Serpine2*	GATCCAAGCGAGGACGGG	GACCTGGATCCCTGTGTTGG	RNAseq validation
*Cd44*	ATCCTCGTCACGTCCAACAC	GCTTTCTGGGGTGCTCTTCT	RNaseq validation
*Jchain*	GACGACGAAGCGACCATTCT	GCTCTGGGTGGCAGTAACAA	RNAseq validation
*Amy2a5*	TTCGTTCTGCTGCTTTCCCT	CATTGGGTGGAGAGACCTGC	RNAseq validation
*Cd7*	ATGCCCAAGACGTACAACAG	CTCCTGCCGGTCTTCAAAGT	RNAseq validation
*Art2a-ps*	GGCTAACCCAGCAGGTGACT	GGCTTCTGTGGATGTTCCCA	RNAseq validation
*Rpl19*	GACCAAGGAAGCACGAAAGC	CAGGCCGCTATGTACAGACA	Reference, RNAseq validation
*Actb*	GACGATGCTCCCCGGGCTGTATTC	TCTCTTGCTCTGGGCCTCGTCACC	Reference, RNAseq validation

### Determination of adenosine levels

The levels of adenosine were determined in the culture supernatants of activated CD4 T cells using the fluorometric adenosine assay kit (Abnova, Walnut, CA) following the methods provided by the manufacturer.

### Statistical analysis

Results are presented as means ± SE, unless otherwise stated. The differences in means between groups were tested using the Studentʼs T-test. Differences in antibody titers were assessed by a 2-way ANOVA. All calculations were made in GraphPad Prism, version 7. A p-value < 0.05 was considered statistically significant. All experiments were performed at least 3 times. *In vivo* experiments consisted of groups of 5 mice and were performed at least twice.

### Ethics statement

All work involving animals was approved by the Institutional Animal Care and Use Committee (IACUC) at CIC bioGUNE and the competent authority (Diputación de Bizkaia). CIC bioGUNE animal facility is accredited by AAALAC Intl. All experiments were performed in accordance with European and Spanish guidelines and regulations.

### Data availability

The transcriptomic data are deposited under GEO accession number GSE98700.

## Electronic supplementary material

Supplementary Information
