# Supplementary material for: The immunosuppressive effect of the tick protein, Salp15, is long-lasting and persists in a murine model of hematopoietic transplant
Source: Sci Rep. 2017 Sep 6;7:10740. doi: 10.1038/s41598-017-11354-2 (PMC5587732; doi:10.1038/s41598-017-11354-2)
Supplement: Supplementary file 1 — Supplementary Information [file 41598_2017_11354_MOESM1_ESM.pdf]

# The immunosuppressive effect of the tick protein, Salp15, is long-lasting and persists in a murine model of hematopoietic transplant

Julen Tomás-Cortázar [1], Itziar Martín-Ruiz [1], Diego Barriales [1], Miguel Ángel Pascual-Itoiz [1], Virginia Gutiérrez de Juan [1], Alfredo Caro-Maldonado [1], Nekane Merino [1], Alberto Marina [1], Francisco J. Blanco [1,2], Juana María Flores [3], James D. Sutherland [1], Rosa Barrio [1], Adriana Rojas [1], María Luz Martínez-Chantar [1,4], Arkaitz Carracedo [1,2,5,6], Carolina Simó [7], Virginia García-Cañas [7], Leticia Abecia [1], José Luis Lavín [1], Ana M. Aransay [1,4], Héctor Rodríguez [1], Juan Anguita [1,2,*]

[1] CIC bioGUNE. 48160 Derio, Bizkaia, Spain.

[2] Ikerbasque, Basque Foundation for Science. 48013 Bilbao, Bizkaia, Spain.

[3] Department of Animal Medicine and Surgery. School of Veterinary Medicine. Complutense University of Madrid. 28040 Madrid, Spain.

[4] Centro de Investigación Biomédica en Red de enfermedades hepáticas y digestivas (CIBERehd) and [5] Centro de Investigación Biomédica en Red en cáncer (CIBERonc), Instituto de Salud Carlos III. 28029 Madrid, Spain.

[6] Department of Biochemistry and Molecular Biology. University of the Basque Country. 48940 Leioa, Bizkaia, Spain.

[7] Molecular Nutrition and Metabolism, Institute of Food Science Research (CIAL, CSIC). 28049 Madrid, Spain.

[*] Correspondence: Juan Anguita, PhD. CIC bioGUNE. Parque Tecnológico de Bizkaia, 48160 Derio, Bizkaia, Spain. +34 944 061 311. janguita@cicbiogune.es

**Supplementary Fig. S1. Validation of the RNAseq on selected genes.** Normalized reads (top) and fold induction changes by qRT-PCR (bottom) of a group of selected genes differentially regulated in CD4 T cells activated with anti-CD3/CD28 for 2 days in the presence of Salp15 (S) or Salp15ΔP11 (C). U: Non-stimulated CD4 T cells.

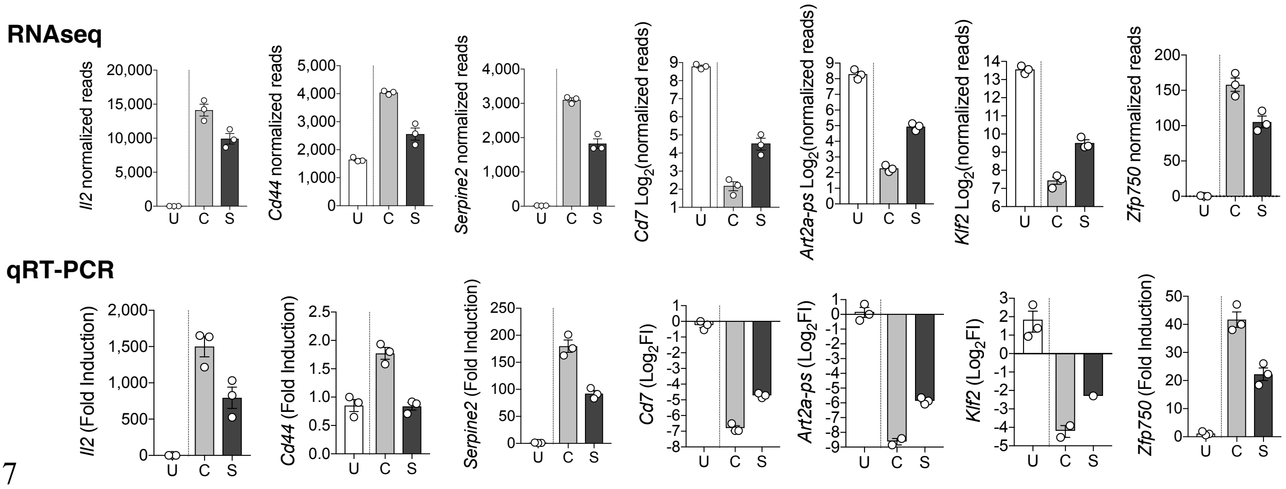

**Supplementary Fig. S2. Gene Ontology analysis of genes affected by Salp15 at 2 days.** Ten most over-represented Gene Ontology of Biological Processes (GOBP) groups affected by Salp15 on activating CD4 T cells. The number of genes (white circles) and the adjusted p values (balck circles) are presented.

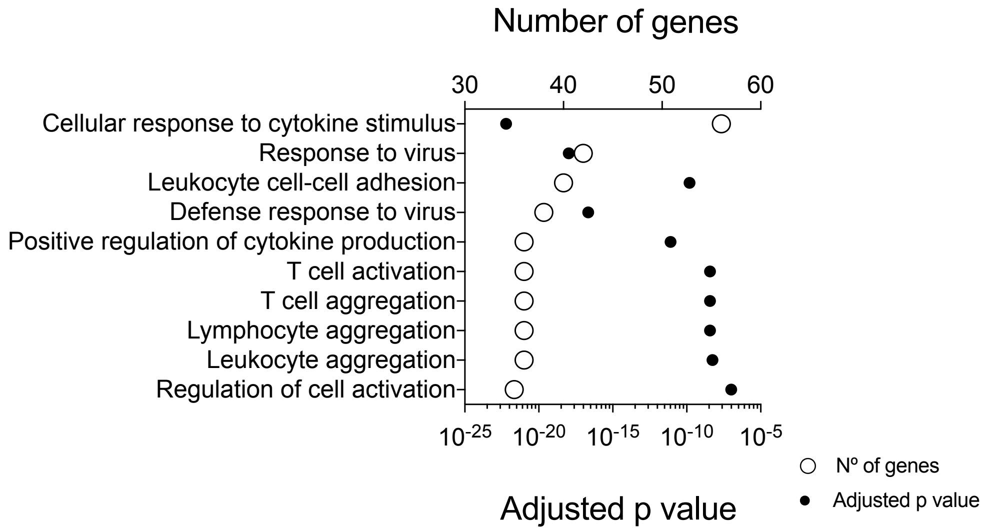

**Supplementary Fig. S3. Gating strategy to identify the populations in ovalbumin-immunized mice.** (A) B cell populations in the blood. (B) CD25 (top right blot), CD44 (bottom left blot) and anergic CD4 T cells (bottom right blot) in the spleen. (C) CD4+NRP1+ (Tregs; top right blot) and myeloid populations in the spleen.

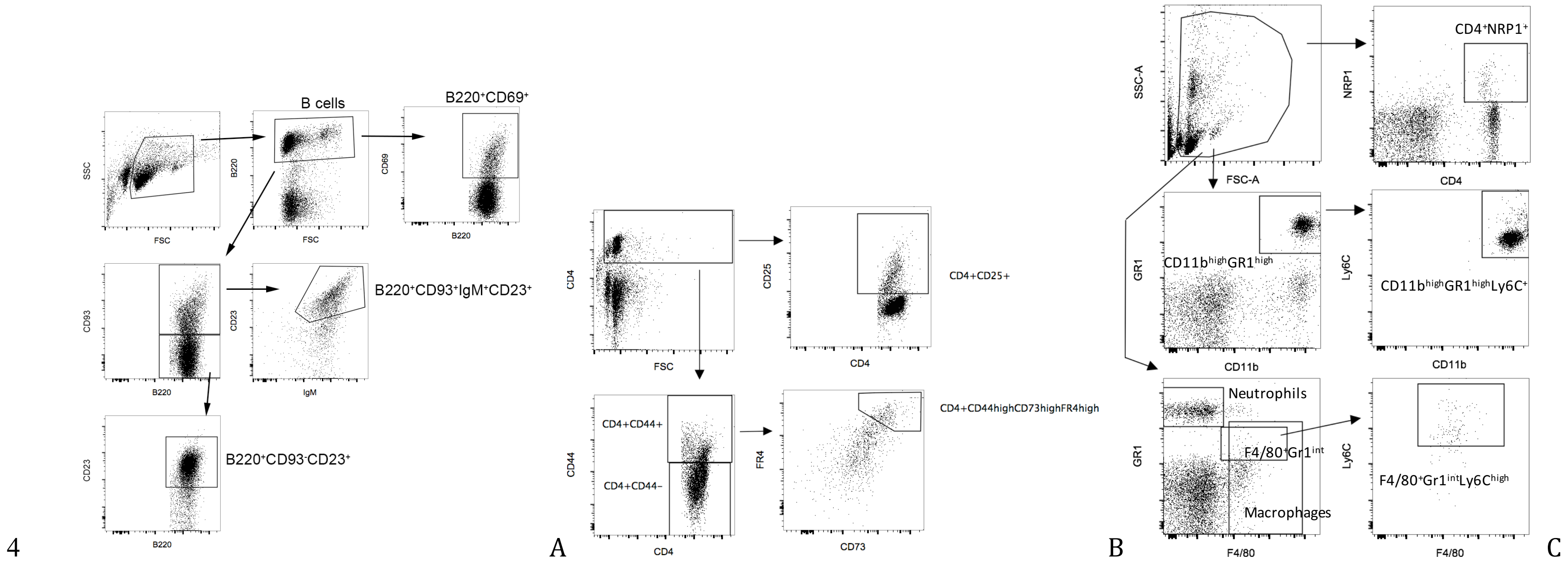

**Supplementary Fig. S4. Salp15 does not affect Th1 or Th2 differentiation in immunized mice.** (A) Heatmap representing the genes associated with CD4 T cell differentiation according to the transcriptomic analysis in the presence of Salp15 (S) or Salp15ΔP11 (C) at 2 and 4 days of activation. (B) Ovalbumin/KLH-immunized mice (see Materials an Methods) were sacrificed at day 21 and whole splenocytes (3 x $10^6$/ml)were restimulated with ovalbumin (Ova) or KLH. The restimulation supernatants were analyzed for IFNγ and IL-4 by capture ELISA. The levels of IFNγ were below the detection limit. No differences were observed between Salp15- or Salp15ΔP11 (control)-treated, immunized mice ($p > 0.05$, 2-way ANOVA). The data represent 10 mice in each group immunized with ovalbumin and 5 per group with KLH and are representative of 2 independent experiments.

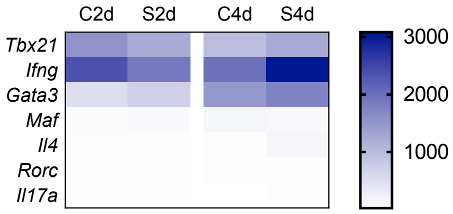


A

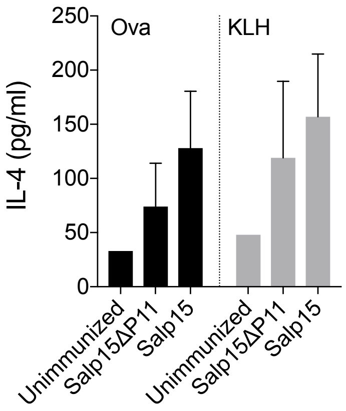


B
